# Inclusivity of the target population in orthopaedic surgical randomised trials: a review of high impact journals

**DOI:** 10.3310/nihropenres.13781.1

**Published:** 2025-01-24

**Authors:** Stephen D Brealey, Lucy Atha, Catherine Knowlson, Elizabeth Cook, Kate Hicks, Joanne Newman, Arabella Scantlebury, Joy Adamson, Caroline Fairhurst, Nick A Johnson, Joseph J Dias

**Affiliations:** 1 Department of Health Sciences, York Trials Unit, University of York, York, England, UK; 2 University Hospitals of Leicester NHS Trust, Leicester, UK

**Keywords:** Inclusivity; Under-served; Orthopaedics; Surgery; Randomised trials; Review

## Abstract

**Background:**

This review examines whether randomised controlled trials (RCTs) of surgery in orthopaedics are inclusive of their target populations, including under-served populations.

**Methods:**

The BMJ, Journal of the American Medical Association, The Lancet, and The New England Journal of Medicine were electronically searched in February 2022 for eligible RCTs published from 1 January 2014. Screening, key baseline patient characteristics, the inclusion of under-served groups and whether patient recruitment was pragmatic in design were key data extracted. The findings were tabulated and reported narratively.

**Results:**

There were 26 RCTs included that were parallel in design and conducted across a range of countries in different hospital settings. Four RCTs did not report the complete CONSORT statement. There was variation in the percentage of the screened population who were randomised into the studies ranging from 5.8% to 74.7%. Most RCTs were pragmatic in design regarding patient selection but this did not necessarily translate to an inclusive trial population. Only two RCTs reported the age and gender of all screened patients. All 26 RCTs reported the age and gender of randomised patients but only four studies reported ethnicity. Reporting about the consideration and inclusion of under-served populations was limited.

**Conclusions:**

There is variation in the exclusion of patients of the target population. Reporting of key patient characteristics during screening and attention given to under-served populations in the design, conduct and reporting of these trials is limited. Training and education on inclusivity is required along with practical guidance about how to implement this. To improve inclusivity in the screening and recruitment of patients there should be a focus on (i) screening and eligibility criteria, (ii) collection and reporting on attributes to ensure no section of the eligible population is inadvertently excluded, and (iii) embedding mechanisms to allow all eligible patients the opportunity to participate.

## Plain Language Summary

Orthopaedic surgical trials often aim to evaluate surgery in real-world settings, including a wide range of participants to reflect the target population. This approach helps ensure the research is relevant to clinical practice and that the findings can be widely applied. The National Institute for Health and Care Research (NIHR) in the United Kingdom is focusing on improving the inclusion of under-represented groups in healthcare research, such as those of different ages, educational backgrounds, and language abilities. Including diverse participants in trials is crucial to avoid missing important findings and to prevent discrimination. This review looked at whether orthopaedic surgical trials are inclusive of their target populations, including under-served groups. It included 26 trials from various countries and hospital settings. The review found that there is variation in how patients are excluded, limited reporting of key patient characteristics, and insufficient attention to under-served populations in the design and conduct of these trials. Training and education on inclusivity is required along with practical guidance about how to implement this. To make trials more inclusive and representative, there should be a focus on the following (i) the criteria for screening and eligibility, (ii) collecting and reporting information to ensure no eligible group is left out, and (iii) create ways for all eligible patients to participate.

## Introduction

Orthopaedic surgical randomised controlled trials (RCTs) are often pragmatically designed to evaluate whether surgery is an effective intervention in a realistic clinical setting ^1, 2^. The evaluation of surgery, lends itself to a pragmatic approach, by its complex nature ^1^. A critical aim of a pragmatic approach is to be inclusive of a broad sample of participants that will reflect the target population in clinical practice and maximise the generalisability of findings. 

Differences in recruitment across sites, however, is common in pragmatic RCTs ^3^. This can result in recruited patients differing from those who are not recruited across characteristics such as age, sex, ethnicity, severity of disease, educational status, social class, and place of residence ^4^. Reviews show that trials consistently fail to report participant flow accurately, particularly before informed consent and randomisation ^5, 6^. As it is not always clear how many patients were screened for inclusion and why they were not randomised, the results may not be accepted by the surgical community. The National Institute for Health and Care Research (NIHR), the United Kingdom’s (UK) largest public funder of trials, has begun to focus on the inclusion of under-represented groups in health care research. Various grouping have been suggested for consideration and include the following: demographic factors (e.g. age, sex, ethnicity); social and economic factors (e.g. employment, socio-economic status, geographic location, language, digitally excluded); and health status (e.g. mental health condition, cognitive impairment, physical disabilities) ^7, 8^. Inclusivity in trials is important for improving representation of the target population so important findings specific to different populations are not missed and to avoid potential discrimination towards historically under-served populations. More money, time and effort may be required to be more inclusive, but may lend itself to research that is representative of the whole patient population and as a result, is more informative for patient and clinical decision-making ^8^. 

For orthopaedic surgical trials, the focus has been on improving internal validity ^1, 9^ rather than on their applicability to practice ^10^. A criticism of such trials has been that the screening, choice, and application of eligibility criteria has meant many patients are excluded. This may affect whether clinicians accept the results of a trial if not considered to be reflective of their usual patients. Treatments may then not be used that could benefit patients and optimise the efficient use of NHS resources, or alternatively, are continued to be used when they have limited clinical and/or cost-effectiveness. This lack of representation has been interpreted as limiting the applicability of the findings of orthopaedic surgical trials ^11, 12^ and can delay their translation into practice and increase research waste ^13^.

In the absence of literature exploring the applicability of orthopaedic surgical trials, that such trials have been criticised for a lack of patient representation, and the emerging policy to be inclusive of underserved groups we judged it was timely to conduct a review on this topic. We chose to focus on orthopaedic trials that are published in high impact medical journals that are likely to have high visibility and potential to influence key stakeholders and clinical practice ^12^. The aim of this review of high impact journals was to examine whether published findings of RCTs of surgery in orthopaedics are inclusive of their target populations and suggest practical recommendations for encouraging inclusivity by design in future orthopaedic surgical trials. 

## Methods

### Patient and Public Involvement

There was no Patient and Public involvement in this research.

We adapted systematic review methodology to robustly review current methodological practice in orthopaedic surgical trials. To optimise study design and transparency in our reporting the protocol and the findings are aligned with the Preferred Reporting Items for Systematic review and Meta-Analysis Protocols (PRISMA-P) checklist and the PRISMA guidance ^14, 15^. The review was prospectively registered with Research on Research hub ( https://ror-hub.org/study/1955/).

### Eligibility criteria

Individually randomised trials that included an orthopaedic patient population defined as involving bone or joint disorders were eligible for inclusion. Trials must have included surgery compared with: other surgical intervention(s); non-operative (i.e. did not involve surgery) interventions; or a placebo-control. Surgery was defined as any interventional procedure that changes the anatomy and requires a skin incision or the use of endoscopic techniques; dental studies were excluded. Placebo refers to a surgical placebo, a sham surgery, or an imitation procedure intended to mimic the active intervention. This includes when a scope is inserted and nothing was done but patients were sedated or under general anaesthesia and could not distinguish whether or not they underwent the actual procedure ^16^. RCTs could be conducted anywhere but only articles written in the English language were included.

### Information sources and search strategy

The BMJ, Journal of the American Medical Association (JAMA), The Lancet, and The New England Journal of Medicine (NEJM) were chosen as examples of top-ranking medical journals and electronically searched in February 2022 for eligible RCTs published from 1 January 2014. That year was chosen as it is when concerns were raised about the DRAFFT trial and the number of patients excluded ^11, 17^. For the BMJ, the date to filter from 01/01/2014 to 06/02/2022 (defaults to today’s date) was selected, “Research” as the type of article and by “trial” in the title. For the Lancet, the publication range could be customised from January 2014 to February 2022. The search terms “randomised” and “trial” were selected to be in the title of “The Lancet” journal and then results selected on Research Article. For JAMA, the search term used was “randomized clinical trial”, then for article type “research” was selected and for content type “article” with a customised date range of 1 January 2014 to 6 February 2022. Finally, for the NEJM, the term “randomized” was used to search within the abstract, “research” for article category and a date range of 2014/01/01 to 2022/02/28). 

### Study selection

One reviewer screened all titles and abstracts to identify potentially eligible studies. Full manuscripts of potentially relevant studies were assessed by the reviewer against the eligibility criteria and independently checked by a second reviewer (LA, CK, EC, KH, JN, JA). Disagreements over eligibility were resolved through discussion or recourse to a third reviewer (SB). 

### Data extraction

A data extraction form was developed in Microsoft Excel and piloted using six studies to assess eligibility and two studies for data extraction. Data collected from the piloting of the form was not included in the review. Data were extracted from the main publication and supplementary files by one reviewer and checked by a second reviewer (LA, CK, EC, KH, JN, JA). Disagreements over data extraction were resolved through discussion or recourse to a third reviewer (SB).

### Data items

Information extracted included author, year, study design (e.g. parallel, factorial), country, setting (e.g. number of trauma hospitals/major trauma centres), target patient population (e.g. top level of the CONSORT ^18^ statement flowchart or equivalent in text), eligibility criteria, recruitment period, intervention/comparator(s), number of patients (screened, excluded, not consented, randomised), reasons for exclusion, recruitment (i.e. where e.g. clinic, ward, intensive care units; how e.g. search medical databases, media advertising, use of incentives) and age (years), gender and ethnicity of patients.

Data extracted about under-served populations and patients being able to consent included: language barriers (e.g. translation, literacy); allowance for disability (e.g. visual/hearing impairment); electronic data collection (i.e. digital disadvantage); and lack of capacity to consent for themselves ^19^.

Domains of the PRECIS-2 tool were used to rate whether recruitment of patients was pragmatic on a scale of 1 to 5 (i.e. very explanatory, rather explanatory, equally pragmatic and explanatory, rather pragmatic, very pragmatic) for eligibility, recruitment and setting ^20^. This was undertaken by one reviewer (NJ) and checked by a second reviewer (JD) and, if necessary, recourse to a third reviewer (SB).

Quality appraisal was not undertaken as it was not an effectiveness review.

### Data synthesis

A narrative and tabular summary of key study characteristics is provided, including the target patient population and eligibility criteria.

The following numbers of patients are presented: (i) screened for enrolment; (ii) excluded based on eligibility criteria; (iii) did not consent; and (iv) randomised. These numbers are presented for all included trials and stratified by the type of comparator.

Age, gender and ethnicity of patients at baseline are summarised descriptively for (i) screened (entire sample); (ii) ineligible (excluded patients); (iii) non-consenting; and (iv) randomised patients. This is presented for all trials and stratified by the type of comparator.

A fixed effects meta-analysis to explore heterogeneity in baseline characteristics (i.e. age, gender, ethnicity) between patients screened but not randomised and those randomised using the I ^2^ statistic was planned ^21^. This was not feasible as there were too few studies, nor for this reason was the planned subgroup analyses about how pragmatic was the trial design or type of comparator.

Finally, whether under-served patient populations were considered including facilitators to consent and whether the trials were pragmatic in the selection and recruitment of patients is tabulated.

## Results

### Study selection

**Figure 1.**
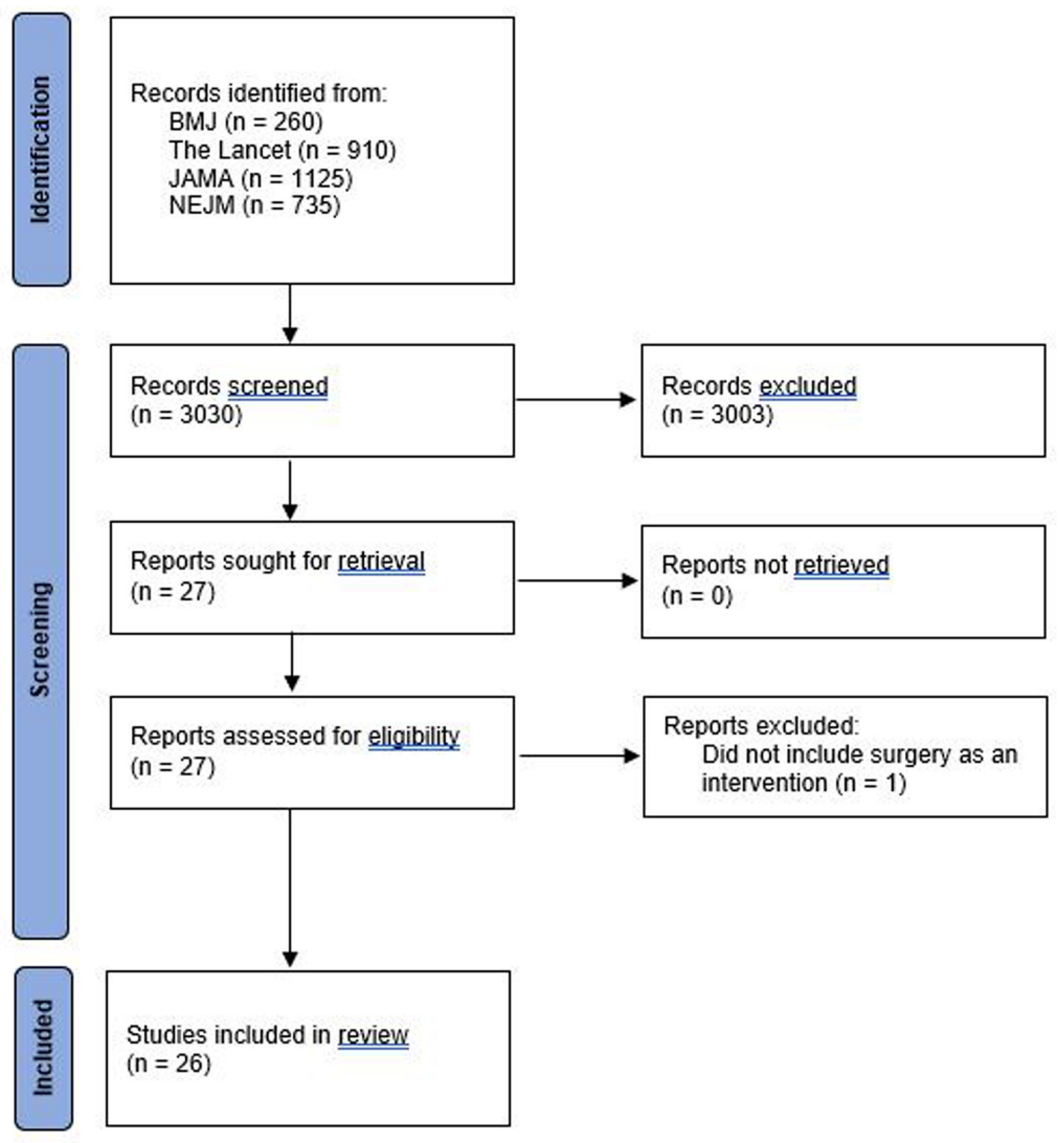
Flowchart of included studies.

3,030 potentially eligible studies were identified. After screening the title and abstract, there was full retrieval of journal articles for 27 studies; one was subsequently excluded that did not include surgery ^22^. Therefore 26 studies were included ^17, 23– 47^. Figure 1 summarises the study selection process. Table 1S in the extended data gives a detailed description of the eligibility criteria and patient population.

### Study characteristics

Table 2S summarises the study characteristics. All studies were parallel in design, four included a sham or placebo-control ^25, 41, 42, 47^, and were conducted across a wide range of countries in different hospital settings. Nine studies did not clearly report where recruitment was undertaken ^17, 23, 24, 32, 34, 36, 37, 39, 47^; among those that did, recruitment took place in locations including out-patient clinics, fracture clinics, wards, and emergency departments. Twelve studies did not report how recruitment was conducted ^17, 23, 24, 28, 31, 33, 34, 39– 42, 47^. For the remaining studies, recruitment included screening by trial co-ordinators/research associates, review by individual or expert panel of surgeons, or new admissions by surgical teams. The detailed eligibility criteria of the included trials are available in Figshare repository for which further details are in the Data Availability section.

### Completion of the CONSORT statement

Table 1 describes the number of patients screened for enrolment, excluded, eligible, non-consenting and randomised as reported in the CONSORT flowchart. Four studies did not fully complete the reporting of patients in the study ^17, 28, 29, 36^. There was variation in the percentage of the screened and eligible population who were randomised ranging from 5.8% up to 74.7% and 30.1% up to 92.4%, respectively. This occurred within studies of different comparators with the surgical intervention.

**Table 1.** CONSORT statement flowchart.

Author (year)	Surgery comparator	Number of patients screened	Number of patients excluded	Number of patients eligible	Number of patients not consenting	Number of patients randomised (% of screened & % of eligible)
Rangan *et al.*, 2020	Multiple comparators	914	116	798	295	503 (55.0% & 63.0%)
Skou *et al.*, 2015	Non-operative intervention	1475	1348	127	27	100 (6.8% & 78.7%)
Bailey *et al.*, 2020	Non-operative intervention	790	622	168	40	128 (16.2% & 76.2%)
Willett *et al.*, 2016	Non-operative intervention	2015	1344	671	51	620 (30.8% & 92.4%)
Rangan *et al.*, 2015	Non-operative intervention	1250	687	563	313	250 (20.0% & 44.4%)
van de Graaf *et al.*, 2018	Non-operative intervention	Not reported	Not reported	Not reported	Not reported	321
Rämö *et al.*, 2020	Non-operative intervention	321	181	140	58	82 (25.5% & 58.6%)
Costa *et al.*, 2022	Non-operative intervention	2636	1936	700	196	504 (19.1% & 72.0%)
Griffin *et al.*, 2014	Non-operative intervention	2006	1504	502	351	151 (7.5% & 30.1%)
Kise *et al.*, 2016	Non-operative intervention	341	115	226	85	140 ^a^ (41.0% & 61.9%)
Palmer *et al.*, 2019	Non-operative intervention	495	145	350	128	222 (44.8% & 63.4%)
Reijman *et al.*, 2021	Non-operative intervention	Not reported	Not reported	282	115	167 (N/A & 59.2%)
Dias *et al.*, 2020	Non-operative intervention	1047	272	775	336	439 (41.9% & 56.6%)
Griffin *et al.*, 2018	Non-operative intervention	6028	5380	648	268 ^b^	348 ^c^ (5.8% & 53.7%)
Ghogawala *et al.*, 2016	Other surgery	Not reported	Not reported	130	64 ^d^	66 (N/A & 50.8%)
Försth *et al.*, 2016	Other surgery	358	59	299	52	247 (69.0% & 82.6%)
Ghogawala *et al.*, 2021	Other surgery	458	168	290	127 ^e^	163 (35.6% & 56.2%)
Costa *et al.*, 2017	Other surgery	537	131	406	85	321 (59.8% & 79.1%)
Costa *et al.*, 2014	Other surgery	Not reported	Not reported	639	178	461 (N/A & 72.1%)
Faith investigators, 2018	Other surgery	7306	5609	1697 ^f^	589	1108 (15.2% & 65.3%)
HIP ATTACK investigators, 2020	Other surgery	27701	19921	7780	532 ^g^	2970 (10.7% & 38.2%)
Beard *et al.*, 2019	Other surgery	962	121	841	310	531 ^h^ (55.2% & 63.1%)
Beard *et al.*, 2018	Placebo control/sham	2975	2235	740	427 ^i^	313 (10.6% & 42.3%)
Paavola *et al.*, 2018	Placebo control/sham	281	68	213	3	210 (74.7% & 98.6%)
Firanescu *et al.*, 2018	Placebo control/sham	1280	944	336	156	180 (14.1% & 53.6%)
Clark *et al.*, 2016	Placebo control/sham	302	148	154	34	120 (39.7% & 77.9%)

^a^ A further patient was not randomised as they incurred another injury following screening ^b ^A further 29 eligible patients were not invited to randomisation consultation ^c ^Three patients were randomised in error and did not receive treatment and were not followed-up ^d ^This is 24 eligible who declined all participation and 40 who declined randomisation but included in an observation cohort ^e ^Of the 127, 91 enrolled into a non-randomised cohort and 15 withdrew prior to randomisation and 21 did not wish to enrol at all/did not wish to have surgery or had surgery at another facility ^f^ The study reports that 1843 were eligible patients; however, of these 146 were potentially eligible but missed so were not confirmed as eligible patients ^g^ A further 4278 were eligible but not randomised for the following reasons: operating room board could not accommodate (n=1643), not identified before surgery (n=1009), surgeon not available (n=396), family did not consent (n=374), physician declined (n=231), other (n=625) ^h^ Of 531, three were randomised twice so excluded ^i^ Of these 427, 232 took part in an observational cohort for patients with a strong preference and 195 did not partake in the trial or cohort

### Description of key baseline characteristics

Tables 3S, 4S and 5S (refer to extended data) describe the key baseline characteristics of age, gender and ethnicity for (i) screened; (ii) ineligible; (iii) non-consenting; and (iv) randomised patients. Only two studies described the characteristics of both screened (age and gender only) and randomised patients ^43, 46^, so heterogeneity was not statistically explored. The same two studies did this for ineligible patients and four studies for non-consenting patients ^17, 36, 43, 46^. All 26 studies reported the age and gender of randomised patients and only four studies for ethnicity ^32, 34, 43, 46^.

### Inclusion of under-served populations

Table 6S (refer to extended data) describes the trial populations for characteristics relevant to under-served populations. There is considerable variation in the choice of lower age limit and ten of the 26 studies (39%) specified an upper age limit (for seven this was ≤75 years) ^24, 25, 28, 29, 32, 33, 36, 39, 42, 44^. Four studies described the ethnicity of trial participants ^32, 34, 43, 46^. Six studies described the education of the trial participants ^28, 29, 33, 40, 43, 46^. Four of those studies, did not describe the entire sample, for example, only whether college education or equivalent was met ^28, 29, 33, 40^. Eight studies reported the employment status of participants ^26, 28, 32, 37, 40, 43, 44, 46^. No studies reported using deprivation scores to help recruit the target population, such as selecting a sample of recruiting sites to reflect a range of geographical populations that are historically under-served by research activity. One study reported including participants living at an alternative place of residence, that is a nursing home ^23^.

### Methods to facilitate consent

Methods to facilitate consent are detailed in Table 7S (refer to extended data). One study reported that language barriers were addressed with the availability of translators ^46^. Two studies reported that study materials were available to potential participants in formats other than written, including the use of a DVD or verbal explanation ^38, 40^. Most studies required written consent and completion of paper questionnaires. No studies reported the use of electronic or verbal consent and only three studies referred to electronic collection of questionnaires ^28, 29, 45^. Few studies mentioned whether patients without capacity to consent were included in their target population (n=7) or patients being excluded for this reason (n=15). Of the remaining four studies, consent was taken by, for example, a legal guardian or was a decision made by the clinical team in the context of the Mental Health Capacity Act 2005 ^23, 30, 34, 36^.

### Pragmatic selection of trial participants

Table 2 summarises how pragmatic studies were in their selection of patients and includes questions about eligibility, recruitment and setting. For eligibility, 19 of the 26 studies (73%) were agreed to be ‘very pragmatic’ or ‘rather pragmatic’ in design, deeming trial participants similar to those patients in usual care. For recruitment, and the extra effort to do this beyond how patients would be identified in usual care, 24 (92%) studies were ‘very pragmatic’ or ‘rather pragmatic’ in design. Then for the setting in which patients were recruited, 16 (62%) studies were ‘very pragmatic’ or ‘rather pragmatic’ in design. 

**Table 2.** Pragmatic selection of patients into the included studies.

Author (year)	**Eligibility—**To what extent are the participants in the trial similar to those who would receive this intervention if it was part of usual care?	**Recruitment—**How much extra effort is made to recruit participants over and above what would be used in the usual care setting to engage with patients?	**Setting—**How different are the settings of the trial from the usual care setting?
Ghogawala *et al.*, 2016	Very explanatory	Rather explanatory	Rather explanatory
Försth *et al.*, 2016	Rather pragmatic	Rather pragmatic	Rather pragmatic
Skou *et al.*, 2015	Equally pragmatic and explanatory	Rather pragmatic	Rather explanatory
Bailey *et al.*, 2020	Rather pragmatic	Rather pragmatic	Very explanatory
Willett *et al.*, 2016	Rather pragmatic	Rather pragmatic	Very pragmatic
Ghogawala *et al.*, 2020	Rather pragmatic	Rather pragmatic	Very pragmatic
Rangan *et al.*, 2015	Very pragmatic	Very pragmatic	Very pragmatic
van de Graaf *et al.*, 2018	Rather pragmatic	Rather pragmatic	Rather pragmatic
Rämö *et al.*, 2020	Very explanatory	Rather pragmatic	Rather explanatory
Costa *et al.*, 2017	Very pragmatic	Very pragmatic	Very pragmatic
Costa *et al.*, 2014	Rather pragmatic	Very pragmatic	Very pragmatic
Costa *et al.*, 2022	Rather pragmatic	Very pragmatic	Very pragmatic
Firanescu *et al.*, 2018	Rather explanatory	Equally pragmatic and explanatory	Equally pragmatic and explanatory
Griffin *et al.*, 2014	Very pragmatic	Very pragmatic	Equally pragmatic and explanatory
Kise *et al.*, 2016	Rather explanatory	Rather pragmatic	Rather explanatory
Paavola *et al.*, 2018	Rather explanatory	Rather pragmatic	Rather explanatory
Palmer *et al.*, 2019	Equally pragmatic and explanatory	Rather pragmatic	Equally pragmatic and explanatory
Reijman *et al.*, 2021	Rather pragmatic	Rather pragmatic	Rather pragmatic
Beard *et al.*, 2018	Rather pragmatic	Very pragmatic	Very pragmatic
Faith investigators, 2018	Very pragmatic	Very pragmatic	Very pragmatic
HIP ATTACK investigators, 2020	Very pragmatic	Rather pragmatic	Very pragmatic
Dias *et al.*, 2020	Very pragmatic	Very pragmatic	Very pragmatic
Beard *et al.*, 2019	Very pragmatic	Very pragmatic	Very pragmatic
Griffin *et al.*, 2018	Rather pragmatic	Rather pragmatic	Rather pragmatic
Rangan *et al.*, 2020	Very pragmatic	Very pragmatic	Very pragmatic
Clark *et al.*, 2016	Rather pragmatic	Rather pragmatic	Very explanatory

## Discussion

This review of orthopaedic surgical trials published in high impact journals illustrates considerable variation in how patients are recruited that could affect clinical applicability and acceptability of trial findings. There is marked variation in patients initially screened, who meet all the eligibility criteria, provide consent and are randomised into the study. Limited data were collected about key baseline characteristics of patients who pass through the different phases of patient selection. Notably only four studies (15%) reported on ethnicity which is similar to a recent review that found only 9.3% (38 of 407) of NIHR trials demonstrated exactly how they both recorded, and reported, ethnicity ^48^. Critical to understanding the selection of patients into RCTs is describing their enrolment in the flowchart of the CONSORT statement ^18^. Most studies reported the different steps of enrolment but there is considerable selectivity of patients from the screened target population to who were randomised into the study. Within included studies we found limited data about ethnicity, education or employment status of patients. The methods did not explain how language barriers were addressed, and what alternative methods of data collection and enrolment of patients without capacity to consent were used. Studies were mostly pragmatic in recruitment of patients, which by definition should have clinical applicability. However, whilst judged to be pragmatic in design ^20^, these findings suggest the need to think beyond what is traditionally considered to be pragmatic and truly be inclusive of all eligible patients and that of under-served populations.

Recently, a lack of patient representation in health care research has become the focus of the NIHR, the largest public funder of trials in the UK. Consequently, there has been an emphasis on including under-served groups. It is known, for example, that for musculoskeletal conditions some minority and ethnic groups are disproportionately represented because of risk factors such as levels of physical activity, vitamin D deficiency, poverty, and pre-existing long term conditions such as diabetes ^49^. A recent national survey from a representative sample of 5,030 people from across the UK found nine in ten people (88%) think a diverse mix of participants in health care and research is important even if the research costs more money (70%) or takes more time (74%) ^50^. Both leading funding bodies and the public expect to have inclusivity in research.

Sometimes trial teams may deliberately widen their screening to ensure every possible patient is considered for the study. Although there may be legitimate reasons for this, several included studies specified an upper age limit of ≤75 years which could proactively exclude eligible patients. When designing studies around the inclusive selection of patients and optimising the flow of patients careful consideration should be given to: defining the target population, the choice of eligibility criteria, who is involved in the screening of patients and the training they have and methods used to screen ^51^, methods to minimise patient and/or surgeon preferences ^52^ and optimise patient recruitment ^53^ and involvement of patient and public collaborators ^54^.

Reporting key characteristics of patients who are screened, excluded because of eligibility criteria and who are not approached or do not consent may help reassure clinicians and policy makers about the representativeness of the trial sample. The General Data Protection Regulation in the UK provides the lawful basis for processing such data and the common law of confidentiality allows the collection of data without a legal basis as long as the patient cannot be identified ^55^. As an example, age could be collected in years (or age bands) or only the first part of a postcode to inform measures of deprivation. This allows the lawful and feasible collection of key characteristics of the screened population without the need for consent. A consistent approach from Research Ethics Committees/Health Research Authority and subsequent Information Governance professionals undertaking local site review is required as to what is acceptable to collect that ensures anonymity but permits reporting about inclusivity. 

In the UK and the NIHR focus on improving inclusivity in research, frameworks are being or have been developed as to how this may be achieved ^7, 8, 56^. This is part of the NIHR workstream called “Innovations in Clinical Trial Design and Delivery for the Under-served” (INCLUDE) project. This includes a roadmap that defines under-served groups and barriers to their inclusion ^8^. The Ethnicity Framework launched on 1 October 2020 ( https://www.trialforge.org/trial-forge-centre/include/) aims to help trial teams think about the inclusion of ethnic groups in their trial ^7^. Multiple approaches to address the barriers to inclusive participation in research include: translation of recruitment and patient questionnaires subject to appropriate validation; and provision of materials in braille, audio-recorded, or animation and apps to help those with low literacy, learning or sensory difficulty. For these tools to be universally adopted into standard trial practice, a coordinated and consistent approach is required to their implementation with a greater understanding of their resource implications to be considerate of the workforce and pressures facing the NHS.

A strength of this review was applying the PRISMA guidelines ^14, 15^. The protocol was registered prospectively. It was conducted by a multi-disciplinary team of methodologists, orthopaedic surgeons and trial coordinators. The review is limited to RCTs of orthopaedic surgical trials in high impact journals. We chose to focus on these RCTs as they are likely to be the best resourced to deliver research and influence key stakeholders and clinical practice. The review team focussed on reporting what was presented in the main publication and supplementary material available on the journal website. It is possible that details not published by the journal are available in a full monograph, in trial registries or in the published protocol; although being described in a registry or protocol does not necessarily mean it was implemented. This review focused on the journal publication and supplementary material as that is most likely to be read by surgeons and to influence decisions in clinical practice. Several reviewers checked study inclusion and undertook data extraction that could contribute to variability in decision-making. This was to make the review feasible with the lack of resources to support it. Maintaining the standard of a second reviewer checking a first reviewer with recourse to always the same third reviewer should mitigate this limitation. Finally, identifying studies with simple search terms were undertaken of the journal website rather than an electronic database such as PUBMED as this was more feasible with the latter lacking specificity in the searches ^57^.

## Conclusion

Patient selection and recruitment is a key challenge for RCTs. Different clinical pathways and differences between participating sites and resources available add to the complexity of achieving this. However, the enrolment of a highly selective sample of patients may impact on the clinical applicability and acceptability of study findings. Trials often purport to be pragmatic in design. The limited data available about who and how patients are included in these studies, questions whether they truly are pragmatic and inclusive of the target population. This review is not a criticism of existing high impact orthopaedic surgical trials that are an important contribution to the evidence-base as only recently has there been this attention towards inclusivity and improving external validity. The challenge now is to address this and ensure every person eligible to take part has the same opportunity and are not excluded whether consciously or not. This is a requirement of leading funding bodies and an expectation of the public. This may be difficult and complex to implement as it requires time, resources and funding for which there can be an opportunity cost and needs to be integrated into efficient trial design and delivery ^58^. Change will also not happen on its own and needs initiatives that provide training and education on inclusivity in clinical trials ^59^ and practical guidance about how to implement strategies to achieve this ^60^. The NIHR is starting initiatives to provide training in inclusive research design ^61^ and regulatory bodies are developing guidance on increasing diversity of people taking part in clinical trials ^62^. The promotion of decentralised clinical trials away from trial sites could also improve inclusivity in recruitment allowing participants to overcome geographical, financial, family and work constraints ^63^. 

Finally, the following practical guidance could improve inclusivity in the screening and recruitment of patients into orthopaedic surgical trials:

(i) screening and eligibility criteria – including collection of data to allow complete reporting of the CONSORT flowchart ^18^, careful consideration in the definition of eligibility criteria ^64^, and efficient data capture methods to record data on those patients screened, eligible, approached and randomised ^51^.

(ii) collection and reporting on attributes to ensure no section of the eligible population is inadvertently excluded – including, for example, collecting data during screening on age, sex, ethnicity and (first part of) postcode to inform measures of deprivation which are often not reported yet known to influence patient outcomes ^4, 65, 66^. 

(iii) embedding mechanisms to allow all eligible patients the opportunity to participate – including making information accessible in a variety of formats such as the translation of recruitment materials; provision of materials in braille, audio-recorded, or animation (that allows captions in different languages); and direction to apps to help read printed materials. 

## Ethics and consent

This study did not require any form of ethical approval or consent

## Data Availability

All data underlying the results are available as part of the article and no additional source data are required. Figshare: Supporting materials for review of high impact journals about inclusivity in orthopaedic surgical randomised trials An additional file including Tables 1S to 7S of extended data is available at Figshare repository along with the trial protocol and PRISMA checklist ( https://doi.org/10.6084/m9.figshare.27074599) ^67^. This project contains the following underlying data: Tables 1S to 7S extended data Applicability of orthopaedic surgical trials review protocol_2023.02.02.docx Figshare: PRISMA checklist for “Inclusivity of the target population in orthopaedic surgical randomised trials: a review of high impact journals”. Doi: https://doi.org/10.6084/m9.figshare.27074599 ^67^ This data is available under the terms of the Creative Commons Zero “No rights reserved” data waiver.
