# Supplementary material for: Inclusivity of the target population in orthopaedic surgical randomised trials: a review of high impact journals
Source: NIHR Open Res. 2025 Nov 13;5:6. Originally published 2025 Jan 24. [Version 4] doi: 10.3310/nihropenres.13781.4 (PMC12640489; doi:10.3310/nihropenres.13781.4)
Supplement: Peer review history

# Reviewer report: Reviewer response for version 3

**DOI:** 10.3310/nihropenres.15349.r37908

**Published:** 2025-11-22

**Authors:** Leica Claydon-Mueller

**Affiliations:** 1 Anglia Ruskin University, Chelmsford, England, UK

This is a narrative literature review which examines whether randomised controlled trials of surgery in orthopaedics in selected high impact journals are inclusive of their target populations, including under-served populations. The publication does not claim to be a systematic review, although reporting guidelines for this design type are referenced and PRISMA flow chart is included.

 The main contribution of this article is the emphasis on the external validity or generalisability of the results of pragmatic randomised controlled trials in orthopaedic surgery which requires increased attention.

Is the work clearly and accurately presented and does it cite the current literature?

Yes

If applicable, is the statistical analysis and its interpretation appropriate?

Not applicable

Are all the source data underlying the results available to ensure full reproducibility?

Yes

Is the study design appropriate and is the work technically sound?

Yes

Are the conclusions drawn adequately supported by the results?

Yes

Are sufficient details of methods and analysis provided to allow replication by others?

Yes

Reviewer Expertise:

literature reviews; systematic review with and without meta-analysis; randomised controlled trials; qualitative research; orthopaedic surgery research

I confirm that I have read this submission and believe that I have an appropriate level of expertise to confirm that it is of an acceptable scientific standard.

# Reviewer report: Reviewer response for version 3

**DOI:** 10.3310/nihropenres.15349.r37161

**Published:** 2025-10-10

**Authors:** Usman Ali

**Affiliations:** 1 Aga Khan University Hospital, Karachi, Pakistan

Thank you for providing your responses to my review. I want to reiterate that the research question you are addressing is both very timely and of high importance. This manuscript has the potential to be a valuable contribution to the literature on inclusivity in clinical trials and orthopedic research.

 My suggestions are intended to help ensure that this important work is presented with the highest level of scientific accuracy and transparency. For the manuscript to realize its potential, several foundational issues of methodological reporting and integrity must be addressed more thoroughly than they have been in the current revision.

 First, and most critically, is the matter of the single-reviewer screening. On a second read, there emerges a significant discrepancy between the limitation I initially identified in my review report and the text that was subsequently added to the Discussion. My concern was the **high risk of selection bias from having a single reviewer who conducted the initial title and abstract screening**. The text added to the Discussion, however, describes a different and unrelated issue: potential "variability in decision-making" caused by having "several reviewers," which links to the later stages of screening without acknowledging the initial bias that might have resulted in the study selection. This does not provide a transparent account of the actual methodological weakness. An honest understanding and acknowledgment of a study's limitations is a strength, not a weakness. The manuscript must be revised to explicitly and accurately state that the initial screening was performed by a single individual and discuss the inherent risk of selection bias this creates. I would highly encourage the authors to strengthen the paper by acknowledging the most important limitations and the impact of those limitations on the manuscript, as this will significantly enhance the transparency and standard of the manuscript.

 Secondly, regarding the classification of the review, the term “adaptation of a systematic review” is not a formally recognized type in established evidence synthesis frameworks like PRISMA, JBI, Cochrane, etc. While I understand the desire to reflect the rigor of your process, using non-standard terminology creates ambiguity for readers and hinders the study's placement within the broader literature. Adhering to standard terminology is crucial for scientific clarity. A more accurate classification, such as a “methodological review” or a “narrative synthesis based on systematic search principles,” should be researched and adopted, with reference to the appropriate guidelines. The chosen classification, with reference to the guiding framework, must be clearly defined in the Methods. If the study does not perfectly align with a single category, this deviation and its rationale should also be detailed in the methodology and acknowledged in the limitations of the manuscript.

 Finally, I appreciate your willingness to add a limitation regarding the journal scope. However, the justification that findings are “unlikely to be different” in specialty journals is speculative without an evidence-based conclusion. More importantly, the impact of this decision on the study's generalizability is profound and cannot be adequately conveyed by a single sentence. This is not a minor point to be listed among other limitations; it is a core boundary defining the applicability of your entire study. Therefore, the manuscript requires a more robust discussion of this limitation, clearly stating that the findings are restricted to a specific subset of the literature and cannot be generalized to the broader orthopaedic field without further research.

 My recommendations for the authors do not call for the review to be re-conducted. My suggestions and findings call for the manuscript to accurately and clearly represent the work that was done, with all its strengths and shortcomings and its undeniable limitations. I am recommending major revisions before this paper can be reconsidered for indexing. A revised manuscript must correct the misrepresentation of the screening limitation, adopt standard terminology for the review type, and provide a robust, transparent discussion of its limited scope.

 I look forward to seeing a substantially revised version that addresses these fundamental points.

Is the work clearly and accurately presented and does it cite the current literature?

Yes

If applicable, is the statistical analysis and its interpretation appropriate?

Yes

Are all the source data underlying the results available to ensure full reproducibility?

Yes

Is the study design appropriate and is the work technically sound?

Partly

Are the conclusions drawn adequately supported by the results?

Yes

Are sufficient details of methods and analysis provided to allow replication by others?

Yes

Reviewer Expertise:

My primary areas of research include orthopaedic research in low- and middle-income countries, health equity, health disparity, and outcomes in underserved communities. This allowed me to assess aspects of this article related to patient inclusivity and representation, and the implications for equitable healthcare

I confirm that I have read this submission and believe that I have an appropriate level of expertise to confirm that it is of an acceptable scientific standard, however I have significant reservations, as outlined above.

# Reviewer report: Reviewer response for version 2

**DOI:** 10.3310/nihropenres.15170.r36215

**Published:** 2025-08-30

**Authors:** Usman Ali

**Affiliations:** 1 Aga Khan University Hospital, Karachi, Pakistan

This is a timely and highly relevant review examining the inclusivity of target populations in orthopaedic surgical RCTs published in high-impact journals. This addresses a critical aspect of clinical trial methodology and generalizability, aligning with contemporary research policy and public interest in diverse representation. The manuscript is generally well-structured, clearly presented, and adheres to key systematic review reporting guidelines, making its methods largely reproducible. One thing of note is the type of study, as the authors mentioned previously that this is not a systematic review. I would suggest expanding on the type of article in the methodology section and clarify what sort of review article it is, if not a systematic review

 Some of the Key strengths of the article are its high relevance, clear and detailed methods, and adherence to reporting standards

 The research question is particularly significant,  addressing a crucial gap in the literature and in understanding patient representation within orthopaedic surgical trials, which has direct implications for clinical applicability and equity in healthcare. The article provides a transparent account of its methodology, including eligibility criteria, data extraction processes, and study selection, which supports the technicality of the work. Although minor limitations are present in the methods section, which are explained below but the overall methodology is robust, clear, and efficient.

**Major Points for Authors' Consideration:**

**Study Selection Methodology and Reviewer Independence:**

 The manuscript states that " *One reviewer screened all titles and abstracts to identify potentially eligible studies. Full manuscripts of potentially relevant studies were assessed by the reviewer against the eligibility criteria and independently checked by a second reviewer*."

 While the full-text assessment involved independent checking by a second reviewer, the initial screening of titles and abstracts was performed by a single reviewer. Standard review practices typically recommend two independent reviewers for both the initial title/abstract screening and the full-text assessment to minimize bias and human error throughout the selection process. Although this approach is sometimes adopted for pragmatic reasons, strengthening this initial step would enhance the robustness of the review.

**Information Sources and Search Strategy, and the Scope of Journals:**

 The review's search strategy was confined to four top-ranking general medical journals (The BMJ, JAMA, The Lancet, and The NEJM). The authors have answered a prior report previously and justified this choice by citing the high visibility and influential nature of these journals, arguing that trials published therein are most likely to inform surgical practice and are well-resourced.

 However, despite this rationale, it remains a significant limitation that the search did not include prominent orthopaedic-specific journals (e.g., JBJS, BJJ, JAAOS, CORR, JOR, Arthroscopy).

**Rationale for Inclusion: **Orthopaedic surgery is a highly specialized field, and a substantial body of relevant, impactful, and prominent orthopaedic surgery related research is exclusively published in specialty journals. The exclusion of these sources risks to potentially missing a large and relevant portion of the orthopaedic surgical trial landscape, which could influence the overall findings on inclusivity.

 Orthopaedic surgeons and residents predominantly rely on orthopaedic specialty journals for most of their focused, clinically relevant knowledge and updates. Specialty journals are highly valued for surgical techniques, subspecialty advances, and research that directly applies to orthopaedic practice. Surveys of orthopaedic residents consistently show that specialty journals like JAAOS and JBJS are considered very important resources for primary scientific literature, clinical updates, and examination studies. (1, 2) 

 While general medical journals such as JAMA or NEJM have broad appeal and are respected for broader medical knowledge, they might be less frequently prioritized compared to orthopaedic-specific journals for day-to-day clinical practice and specialized research. A review of orthopaedic trials should ideally reflect the literature directly accessed by the orthopaedic community. Also the findings, while valuable for trials in high-impact general journals, may not fully represent or generalize to the broader spectrum of orthopaedic surgical trials, which often appear in dedicated specialty publications.

 I would urge the authors to reconsider the implications of this scope limitation more deeply, perhaps by acknowledging that their "snapshot" primarily reflects inclusivity in orthopaedic trials published in general medical journals, rather than the full breadth of orthopaedic surgical research.

 If the authors are unable to re-conduct the search to include orthopaedic-specific journals, I strongly recommend they enhance their acknowledgment of this limitation. This can be achieved by explicitly stating in the 'Limitations' section that the findings are primarily generalizable to orthopaedic surgical RCTs published in high-impact general medical journals, and may not fully reflect practices or issues prevalent in trials published within orthopaedic journals. Furthermore, throughout the 'Discussion' and 'Conclusion' sections, statements should be consistently qualified to remind the reader of this specific journal scope, for instance, by stating 'orthopaedic surgical trials published in high-impact general medical journals often demonstrate limitations in inclusivity.' Finally, when discussing implications for orthopaedic practice, it should be acknowledged that direct applicability might be primarily for surgeons who rely more on general medical literature, rather than those who primarily consult specialty orthopaedic journals for updates. This consistent and explicit framing will prevent misinterpretation and clearly delineate the boundaries of the review's generalizability; however, I strongly recommend the inclusion of orthopaedic-specific journals. Given the excellence and timeliness of the research question, the authors should not miss the opportunity to incorporate such valuable information for a truly comprehensive understanding of inclusivity in orthopaedic surgical research.

**Coherence of the Discussion Section:**

 The first paragraph of the Discussion effectively summarizes the direct findings of the review, outlining variations in recruitment and limitations in baseline characteristic reporting.

 However, the subsequent paragraphs sometimes lean towards a more general narrative about inclusivity policies (e.g., NIHR initiatives, public opinion) and broad recommendations for trial design. While these elements are crucial for contextualizing the findings and providing a path forward, a clearer and more explicit link back to the specific aggregated findings from the screened articles could be maintained throughout the discussion. This would help ensure that the entire discussion remains firmly anchored in the review's empirical results and avoids appearing overly generalized.

 I thank the Journal for the opportunity to review this manuscript. The authors are to be commended for identifying such a pertinent and interesting topic within orthopaedic surgical research and for undertaking this review. Please note that the points raised in this report reflect my personal assessment and professional opinion, which may, of course, differ from those who are experts in the field.

Is the work clearly and accurately presented and does it cite the current literature?

Yes

If applicable, is the statistical analysis and its interpretation appropriate?

Yes

Are all the source data underlying the results available to ensure full reproducibility?

Yes

Is the study design appropriate and is the work technically sound?

Partly

Are the conclusions drawn adequately supported by the results?

Yes

Are sufficient details of methods and analysis provided to allow replication by others?

Yes

Reviewer Expertise:

My primary areas of research include orthopaedic research in low- and middle-income countries, health equity, health disparity, and orthopaedics in underserved communities. This allow me to particularly assess aspects of this article related to patient inclusivity and representation, and the implications for equitable healthcare

I confirm that I have read this submission and believe that I have an appropriate level of expertise to confirm that it is of an acceptable scientific standard, however I have significant reservations, as outlined above.

## References

[1] : Educational resource utilization by current orthopaedic surgical residents: a nation-wide survey. JAAOS Global Research &.2019;10.5435/JAAOSGlobal-D-19-00041PMC651046231334477

[2] : Orthopedic surgery residents' study habits and performance on the orthopedic in-training examination. Am J Orthop.2007;18264562

# Response

**Published:** 2025-09-12

**Authors:** Stephen Brealey

**Affiliations:** University of York Department of Health Sciences, York, England, UK

This is a timely and highly relevant review examining the inclusivity of target populations in orthopaedic surgical RCTs published in high-impact journals. This addresses a critical aspect of clinical trial methodology and generalizability, aligning with contemporary research policy and public interest in diverse representation. The manuscript is generally well-structured, clearly presented, and adheres to key systematic review reporting guidelines, making its methods largely reproducible. One thing of note is the type of study, as the authors mentioned previously that this is not a systematic review. I would suggest expanding on the type of article in the methodology section and clarify what sort of review article it is, if not a systematic review.

***Response: **Thank you for your thoughtful and constructive comments about our review; we are grateful to have the opportunity to respond. The study design could be described as a “narrative review” of the literature, which was conducted as robustly as was feasible by adapting key methodological principles from systematic reviews with the resources available. It wasn’t an effectiveness review, which allowed a more flexible application of systematic review methodology. However, characterising this study as a “narrative review” would not fully represent the methodological rigour undertaken. Given the extensive adaptations of systematic review methodology to enhance the study's robustness, we prefer to keep to the current description of the review in the Methods section that is both accurate and appropriate.*

 Some of the Key strengths of the article are its high relevance, clear and detailed methods, and adherence to reporting standards

 The research question is particularly significant,  addressing a crucial gap in the literature and in understanding patient representation within orthopaedic surgical trials, which has direct implications for clinical applicability and equity in healthcare. The article provides a transparent account of its methodology, including eligibility criteria, data extraction processes, and study selection, which supports the technicality of the work. Although minor limitations are present in the methods section, which are explained below but the overall methodology is robust, clear, and efficient.

**Major Points for Authors' Consideration:**

**Study Selection Methodology and Reviewer Independence:**

 The manuscript states that " *One reviewer screened all titles and abstracts to identify potentially eligible studies. Full manuscripts of potentially relevant studies were assessed by the reviewer against the eligibility criteria and independently checked by a second reviewer*."

 While the full-text assessment involved independent checking by a second reviewer, the initial screening of titles and abstracts was performed by a single reviewer. Standard review practices typically recommend two independent reviewers for both the initial title/abstract screening and the full-text assessment to minimize bias and human error throughout the selection process. Although this approach is sometimes adopted for pragmatic reasons, strengthening this initial step would enhance the robustness of the review.

***Response: **We agree that a more robust study selection process would have enhanced its rigour. However, as explained in the Discussion, a more pragmatic approach was taken with the limited resources available to complete this review. It is unlikely these additional steps would alter the clear message found from this review. Nonetheless, to acknowledge this limitation, it has been added to the Discussion.*

**Information Sources and Search Strategy, and the Scope of Journals:**

 The review's search strategy was confined to four top-ranking general medical journals (The BMJ, JAMA, The Lancet, and The NEJM). The authors have answered a prior report previously and justified this choice by citing the high visibility and influential nature of these journals, arguing that trials published therein are most likely to inform surgical practice and are well-resourced.

 However, despite this rationale, it remains a significant limitation that the search did not include prominent orthopaedic-specific journals (e.g., JBJS, BJJ, JAAOS, CORR, JOR, Arthroscopy).

**Rationale for Inclusion: **Orthopaedic surgery is a highly specialized field, and a substantial body of relevant, impactful, and prominent orthopaedic surgery related research is exclusively published in specialty journals. The exclusion of these sources risks to potentially missing a large and relevant portion of the orthopaedic surgical trial landscape, which could influence the overall findings on inclusivity.

 Orthopaedic surgeons and residents predominantly rely on orthopaedic specialty journals for most of their focused, clinically relevant knowledge and updates. Specialty journals are highly valued for surgical techniques, subspecialty advances, and research that directly applies to orthopaedic practice. Surveys of orthopaedic residents consistently show that specialty journals like JAAOS and JBJS are considered very important resources for primary scientific literature, clinical updates, and examination studies. (1, 2) 

 While general medical journals such as JAMA or NEJM have broad appeal and are respected for broader medical knowledge, they might be less frequently prioritized compared to orthopaedic-specific journals for day-to-day clinical practice and specialized research. A review of orthopaedic trials should ideally reflect the literature directly accessed by the orthopaedic community. Also the findings, while valuable for trials in high-impact general journals, may not fully represent or generalize to the broader spectrum of orthopaedic surgical trials, which often appear in dedicated specialty publications.

 I would urge the authors to reconsider the implications of this scope limitation more deeply, perhaps by acknowledging that their "snapshot" primarily reflects inclusivity in orthopaedic trials published in general medical journals, rather than the full breadth of orthopaedic surgical research.

 If the authors are unable to re-conduct the search to include orthopaedic-specific journals, I strongly recommend they enhance their acknowledgment of this limitation. This can be achieved by explicitly stating in the 'Limitations' section that the findings are primarily generalizable to orthopaedic surgical RCTs published in high-impact general medical journals, and may not fully reflect practices or issues prevalent in trials published within orthopaedic journals. Furthermore, throughout the 'Discussion' and 'Conclusion' sections, statements should be consistently qualified to remind the reader of this specific journal scope, for instance, by stating 'orthopaedic surgical trials published in high-impact general medical journals often demonstrate limitations in inclusivity.' Finally, when discussing implications for orthopaedic practice, it should be acknowledged that direct applicability might be primarily for surgeons who rely more on general medical literature, rather than those who primarily consult specialty orthopaedic journals for updates. This consistent and explicit framing will prevent misinterpretation and clearly delineate the boundaries of the review's generalizability; however, I strongly recommend the inclusion of orthopaedic-specific journals. Given the excellence and timeliness of the research question, the authors should not miss the opportunity to incorporate such valuable information for a truly comprehensive understanding of inclusivity in orthopaedic surgical research.

*Response: We focussed on high-impact journals for the reasons given in the publication. We understand that surgeons will consider speciality journals for day-to-day practice. However, policy and practice, such as that informed by NICE guidance, will be very much influenced by large-scale well-funded clinical trials. We argue that if well-funded studies with teams of collaborators across multiple institutions and sometimes across multiple countries do not appropriately address inclusivity it is unlikely that this will be different for evidence published in specialty journals. It was not feasible to include evidence from orthopaedic-specific journals. We have, as has been suggested, emphasised the limited scope and generalisability of the review in the Discussion.*

**Coherence of the Discussion Section:**

 The first paragraph of the Discussion effectively summarizes the direct findings of the review, outlining variations in recruitment and limitations in baseline characteristic reporting.

 However, the subsequent paragraphs sometimes lean towards a more general narrative about inclusivity policies (e.g., NIHR initiatives, public opinion) and broad recommendations for trial design. While these elements are crucial for contextualizing the findings and providing a path forward, a clearer and more explicit link back to the specific aggregated findings from the screened articles could be maintained throughout the discussion. This would help ensure that the entire discussion remains firmly anchored in the review's empirical results and avoids appearing overly generalized.

*Response: The authors have carefully considered this comment. The Discussion is anchored to the evidence found in this review and has been discussed in the context of the current guidance and literature on this topic to optimise inclusivity into trials. Notably the Discussion ends with practical guidance to improve inclusivity into orthopaedic surgical trials. Therefore, no further edits to the Discussion are considered necessary.*

 I thank the Journal for the opportunity to review this manuscript. The authors are to be commended for identifying such a pertinent and interesting topic within orthopaedic surgical research and for undertaking this review. Please note that the points raised in this report reflect my personal assessment and professional opinion, which may, of course, differ from those who are experts in the field.

# Reviewer report: Reviewer response for version 2

**DOI:** 10.3310/nihropenres.15170.r35122

**Published:** 2025-06-12

**Authors:** Ameeta Retzer

**Affiliations:** 1 University of Birmingham, Birmingham, England, UK

Thank you for addressing the comments I had previously outlined. I have no further comments.

Is the work clearly and accurately presented and does it cite the current literature?

Yes

If applicable, is the statistical analysis and its interpretation appropriate?

Not applicable

Are all the source data underlying the results available to ensure full reproducibility?

No source data required

Is the study design appropriate and is the work technically sound?

Partly

Are the conclusions drawn adequately supported by the results?

Yes

Are sufficient details of methods and analysis provided to allow replication by others?

Partly

Reviewer Expertise:

- systematic evaluation of clinical trial protocols and reports, inclusive and equitable research design. I am not an expert in surgical or orthopaedic research.

I confirm that I have read this submission and believe that I have an appropriate level of expertise to confirm that it is of an acceptable scientific standard.

# Reviewer report: Reviewer response for version 1

**DOI:** 10.3310/nihropenres.14969.r34610

**Published:** 2025-03-03

**Authors:** Kim Madden

**Affiliations:** 1 McMaster University, Hamilton, Ontario, Canada

Thank you for the opportunity to review this paper. Inclusivity is a key issue in orthopaedics and the topic is very important. However, I have several comments about the paper. 

 1. Several studies have been missed. There are more than 26 orthopaedic trials in major journals in the past decade. E.g. [Ref 1] [Ref 2].

  The search strategy is flawed because the exact term "randomized" is not always used. Sometimes it is "randomly assigned" or "randomization" as in the examples given here.

 2. This type of analysis that involves describing the excluded sample is challenging because each site or local ethics committee will have different requirements/processes for screening and recording who was screened. Our site is not allowed to keep any data on patients who do not consent. Researchers in my jurisdiction are not even allowed to know about patients who declined to be screened or declined to talk to research staff so those are never reported. I have noticed vastly different numbers of patients screened at different sites, not because of different actual numbers of patients, but different screening processes and privacy laws.

 3. Why use a fixed effects model? Random effects seems more appropriate here.

 4. "One study reported including participants living at an alternative place of residence, that is a nursing home [cites the HIP ATTACK trial]". The HIP ATTACK trial didn't recruit from nursing homes, it included patients presenting to level 1 trauma centres from anywhere, including nursing homes. Many hip fracture trials would do the same. This is a bit misleading and could be worded better. Something like "one study reported on place of residence (e.g. independent or nursing home).

Is the work clearly and accurately presented and does it cite the current literature?

Yes

If applicable, is the statistical analysis and its interpretation appropriate?

Partly

Are all the source data underlying the results available to ensure full reproducibility?

Partly

Is the study design appropriate and is the work technically sound?

Partly

Are the conclusions drawn adequately supported by the results?

Partly

Are sufficient details of methods and analysis provided to allow replication by others?

Partly

Reviewer Expertise:

research methodology, clinical trials, systematic reviews, orthopaedic trauma, arthroplasty

I confirm that I have read this submission and believe that I have an appropriate level of expertise to state that I do not consider it to be of an acceptable scientific standard, for reasons outlined above.

## References

[1] : Total Hip Arthroplasty or Hemiarthroplasty for Hip Fracture. *N Engl J Med* .2019;381(23) : 10.1056/NEJMoa1906190 2199-2208 10.1056/NEJMoa1906190 31557429

[2] : A Trial of Wound Irrigation in the Initial Management of Open Fracture Wounds. *N Engl J Med* .2015;373(27) : 10.1056/NEJMoa1508502 2629-41 10.1056/NEJMoa1508502 26448371

# Response

**Published:** 2025-03-31

**Authors:** Stephen Brealey

**Affiliations:** University of York Department of Health Sciences, York, England, UK

Thank you for your comments on our article.

 1. We comment in the last paragraph of the Discussion that it was more feasible to search by journal and we adapted the terms we used specific to the journal being searched.

 2. We comment in the fourth paragraph of the Discussion that a consistent approach is required by committees to allow collection of these data and what is agreed locally by IG professionals. We have added an extra sentence to this paragraph to recognise your important point about how this data capture may be limited by different jurisdictions.

 3. The planned meta-analysis to explore heterogeneity in baseline characteristics (i.e. age, gender, ethnicity) between patients screened but not randomised and those randomised was not ultimately feasible as there were too few studies.  This analysis was planned to explore the assumption that if the trials were truly inclusive then the participants randomised would be a representative sample of those screened, and as such there should be no difference between the baseline characteristics of these two groups, rather like if they had been formed by random allocation. Therefore, a fixed effects meta-analysis was planned rather than a random effects on the assumption that there was a common treatment estimate (i.e. zero) for the baseline characteristics between these two groups across the trials and the only legitimate source of between study variation is due to chance, which is accounted for in a fixed effects model.  Whereas a random effects model assumes and allows for heterogeneity i.e. in this case that the difference between the baseline characteristics of the two groups would vary across the different trials.  Following the fixed effects meta-analysis, we would have interpreted the resulting I ^2 ^value of heterogeneity in line with the Cochrane handbook guidelines (i.e. 0%–40% might not be important; 30%–60% may represent moderate heterogeneity; 50%–90% may represent substantial heterogeneity; and 75%–100% considerable heterogeneity).

 Ref. Hicks A, Fairhurst C, Torgerson DJ. A simple technique investigating baseline heterogeneity helped to eliminate potential bias in meta-analyses. *J Clin Epidemiol*. 2018;95:55-62.

 4. Thank you, we have modified the sentence as suggested.

 We understand your concerns about potentially missing some studies, however, our aim was not to undertake a systematic review like that for effectiveness. For the reasons given, we purposefully focused on high impact journals and to keep it feasible with no funding. We have undertaken a comprehensive review and used the PRISMA principles where appropriate to bring rigour to its design and conduct. Inclusivity is an important topic and our take home message is that, even among the highest quality journals, it is difficult for surgeons to make an assessment of whether a trial population is representative of their clinical population. We think this is an important message that would not have been impacted by the methods selected. Therefore, in our opinion, this remains an original and timely contribution to the literature.

# Reviewer report: Reviewer response for version 1

**DOI:** 10.3310/nihropenres.14969.r34609

**Published:** 2025-02-18

**Authors:** Ameeta Retzer

**Affiliations:** 1 University of Birmingham, Birmingham, England, UK

1. This article describes a review of randomised controlled trials in orthopaedic surgery and whether they are inclusive of their target populations, including under-served groups. This is a timely and interesting study of research inclusion in orthopaedic surgery.

 2. The article is clearly presented and appears to cite the appropriate literature. 

 3. Study design and description of methods - I am not familiar with the methods used to identify the literature through searching journal databases. In the discussion, Hopewell et al are cited for their study on using handsearching versus electronic searching to identify RCT reports to justify use of this method over the use of electronic databases. This reference is from 2007 and I am not sure if this justification still stands as the usual practice to systematically identify literature. While the journals searched are high-impact, it is not clear on what basis these were chosen and it may be that surgery-specific journals or orthopaedics journals would have been more appropriate. I am also not familiar with how journals index their articles so not clear on whether the terms used were appropriate. I would suggest expanding on this in the limitations section of the paper as the methods as they are currently described and justified do not assure that this was the most systematic way to identify the relevant literature to address this research question. Further due to the variation in journalistic styles of each of the journals, there is likely to be variation in the volume of detail in each of the articles e.g one journal may have a more restrictive wordcount than another or a policy of sharing the protocol as a supplementary appendix may have been introduced as a different time. As such, the comparison between two articles from different journals may not be like with like nor would they necessarily yield the necessary information to answer the research question.

Is the work clearly and accurately presented and does it cite the current literature?

Yes

If applicable, is the statistical analysis and its interpretation appropriate?

Not applicable

Are all the source data underlying the results available to ensure full reproducibility?

No source data required

Is the study design appropriate and is the work technically sound?

Partly

Are the conclusions drawn adequately supported by the results?

Yes

Are sufficient details of methods and analysis provided to allow replication by others?

Partly

Reviewer Expertise:

- systematic evaluation of clinical trial protocols and reports, inclusive and equitable research design. I am not an expert in surgical or orthopaedic research.

I confirm that I have read this submission and believe that I have an appropriate level of expertise to confirm that it is of an acceptable scientific standard, however I have significant reservations, as outlined above.

# Response

**Published:** 2025-03-31

**Authors:** Stephen Brealey

**Affiliations:** University of York Department of Health Sciences, York, England, UK

Thank you for your comments on our article. We agree that the method of handsearching has limitations, however, this is not a systematic review like would be done to assess effectiveness, and the focus on specific journals meant we chose to search them directly. The final paragraph of the Introduction and the Discussion explains why we focus on high impact journals rather than surgery-specific journals i.e. these trials are most likely to inform surgical practice and to be the most resourced to address all the aspects of inclusivity of the target population. We chose the specific journals as they are amongst the most cited medical journals and to the authors of this review, they are journals known to have published large scale surgical trials that are likely to influence practice. Similarly, we acknowledge in the last paragraph of the Discussion why we focused on the journal article and Supplementary material, again this is what is likely to be read by surgeons to inform their practice. Journals will have different policies about word count and the extent to which material is available; this, however, is what surgeons access to inform decision-making. We have elaborated a little further on these points in the final paragraph of the Discussion.

 Essentially, we were trying to provide a ‘snapshot’ of what could be considered best practice in trial publication, in order to highlight issues relating to inclusivity. We do not expect that wider inclusion criteria would have changed the findings from this review. What we have learned is that, based on trials published in, arguably, the most prestigious and influential journals, it is difficult for clinicians to assess whether the characteristics of those participating in the trial reflects the demographics of the patient population. We think this is an important finding to make known to the research community.
